# Supplementary material for: Modeling reveals a metabolic basis of competition among Dehalobacter strains during tandem chloroform and dichloromethane metabolism
Source: mSystems. 2025 Sep 5;10(10):e00847-25. doi: 10.1128/msystems.00847-25 (PMC12542780; doi:10.1128/msystems.00847-25)
Supplement: Supplemental material — Supplemental texts and figures, links to external data sets, and Tables S1-S7. [file msystems.00847-25-s0001.pdf]

Supplementary Information

Modeling reveals metabolic basis of competition among *Dehalobacter* strains during tandem chloroform and dichloromethane metabolism

Olivia Bulka[1], Elizabeth A. Edwards[1], and Radhakrishnan Mahadevan[1*].

[1]Department of Chemical Engineering and Applied Chemistry, University of Toronto, Toronto, Ontario M5S 3E5, Canada.

*Corresponding Author: Radhakrishnan Mahadevan, e-mail: krishna.mahadevan@utoronto.ca

Table of Contents

External Datasets 2

Supplementary Tables 3

Supplementary Figures 4

1. Model Construction 5
   - 1.2. Biomass equation 5
   - 1.3. Theoretical maximum energy transfer efficiency and proton translocation stoichiometry of *Dehalobacter* electron transport chain 7
   - 1.4. Calculation of maintenance parameters 11
   - 1.5. Validation of thermodynamic constraints compared to experimental data 11
2. Genomic curation 12
   - 2.1. Curation of energy metabolism 16
   - 2.2. DCM metabolism and the Wood-Ljungdahl pathway 20
   - 2.3. TCA cycle and amino acid metabolism 20
   - 2.4. Vitamins and cofactors 20
3. Flux balance analysis in *Dehalobacter* strains SAD and DAD 22
   - 3.1. Abbreviation key (reactions and metabolites) 22
   - 3.2. The impact of product inhibition on H_2-evolving hydrogenases 26
   - 3.3. Yield validation of different modes 28
   - 3.4. Role of methanogens in SC05 28

References for SI 30

External Datasets

All external datasets are deposited as follows:

Dataset S1. All detected genes involved in anaerobic cobalamin biosynthesis in the SC05-UT metagenome at JAWDGN000000000, and their predicted taxonomy.
DOI:10.6084/m9.figshare.26972683

Dataset S2. Computational and statistical analysis of peptides and proteins from liquid chromatography tandem mass spectrometry (LC-MS/MS) output. This table contains all values from LC-MS/MS read analysis including X!Tandem algorithm intensity score and identification of peptides and proteins.
DOI:10.6084/m9.figshare.26972656

Models. The models of *Dehalobacter* strains SAD and DAD, and a jupyter notebook containing the code for FBA simulations are available on GitHub.
https://github.com/LMSE/Dehalobacter_modelling

Supplementary Tables

Table S1. Standard free energies of formation 8

Table S2. Half reactions (as reductions) and their Gibb's standard free energy 9

Table S3. Selection of theoretical thermodynamic constraints for energy metabolism of *Dehalobacter*. .10

Table S4. Reactions added to each *Dehalobacter* metabolic model based on genome presence. NA: not applicable (gene-free reaction), ND: not detected. 12

Table S5. Genomic localization and proteomic expression of key metabolic reactions in *Dehalobacter* strains SAD and DAD. 14

Table S6. Classification of hydrogenases in each *Dehalobacter* strain, and cofactors encoded downstream of the catalytic gene and predicted by HYDdb...17

Table S7. Summary of key genera encoding genes molybdopterin biosynthesis PFAM/TIGRFAM motifs in the SC05-UT metagenome. 19

Table S8. Cofactor and vitamin biosynthesis pathways in each species, and supplementation in mineral medium; Y: present, N: absent. 21

Table S9. Proteins identified using LC-MS/MS from the SC05-UT and DCME enrichment cultures that map to *Dehalobacter* strains SAD and DAD. ....*in supplementary excel file*

Table S10. Description of each simulation case. ....*in supplementary excel file*

Table S11. All fluxes predicted by FBA optimization of metabolic models.......*in supplementary excel file*

Table S12. Summary of growth rate, ATP production, and export/uptake of key metabolites and amino acids ....*in supplementary excel file*

Supplementary Figures

Figure S1. Impact of biomass composition on yield in *Dehalobacter* models 7

Figure S2. Alignment of key metabolic gene differences in *Dehalobacter* strains SAD and DAD 13

Figure S3. Alignment of hydrogenase gene clusters in in *Dehalobacter* strains SAD and DAD and their predicted classifications 18

Figure S4. Flux distribution during growth **Mode 3, Strategy A**, using DCM as an electron donor and H^+ as an electron acceptor, with minimal amino acid supplementation. 23

Figure S5. Flux distribution during growth **Mode 3, Strategy B**, using DCM as an electron donor and H^+ as an electron acceptor, with addition of an NADH-dependent hydrogenase HYD-NADH. 24

Figure S6. Flux distribution during growth **Mode 3, Strategy C**, using DCM as an electron donor and H^+ as an electron acceptor, with addition of an NADH-dependent hydrogenase HYD-NADH and minimal amino acid supplementation. 25

Figure S7. Flux distribution during growth **Mode 1, Strategy A, H_2 evolution inhibition**, using H_2 as an electron donor and CF as an electron acceptor 27

Figure S8. Thermodynamics of CF dechlorination and methanogenesis at various H_2 concentrations. 29

1. Model Construction

1.1. Genome-scale metabolic model structure

Each model consists of a stoichiometric matrix S ($m \times n$), where m is the number of metabolites and n is the number of reactions. Each entry in the stoichiometric matrix (S_{ij}) represents the stoichiometric coefficient for the ith metabolite in the jth reaction. Linear programming can be used to solve for v ($n \times 1$)—a vector of reaction fluxes—assuming steady state.

$$Sv = 0$$

(Eq. 1)

To solve for v, the objective function is set to maximize flux through the biomass synthesis reaction ($v_{biomass}$), further described in Section 1.2, and reactions are constrained to:

$$lb_i \leq v_i \leq ub_i$$

(Eq. 2)

where lb = lower bound

ub = upper bound

for reversible reactions, fluxes are constrained as $-1000 \leq v_i \leq 1000$

for irreversible reactions, fluxes are constrained to $0 \leq v_i \leq 1000$ or $-1000 \leq v_i \leq 0$, depending on directionality.

The biomass reaction includes all components required for one gram of dry weight (gdw), as well as growth associated maintenance (GAM), in the form of ATP hydrolysis, to account for nonmetabolic growth activity. The non-growth associated maintenance (NGAM) is represented as a set flux through an ATP hydrolysis reaction (the flux vector, v_{NGAM}). The GAM and NGAM parameter estimation is discussed in Section G.4.

The three metabolic modes were simulated with chloroform (CF, EX_cf_e), dichloromethane (DCM, EX_dcm_e), and hydrogen (EX_h2_e) uptake constrained as follows, in mmol gdw^{-1} d^{-1}.

Mode 1: CF, –10; DCM, 0; H_2, –10.

Mode 2: CF, –10; DCM, 0; H_2, 0.

Mode 3: CF, 0; DCM, –10; H_2, 0.

1.2. Biomass equation

The biomass composition is representative of slow-growing microbes (1): 50% protein, 10% RNA, 5% DNA, 5% phospholipids, 25% peptidoglycan, 5% ash (inorganic compounds). The amino acid composition of *Dehalobacter* protein was estimated using measured values from another gram positive

organism, *Bacillus subtilis* (mmol/g protein) (2): L-alanine, 1.2437; L-arginine, 0.3646; L-asparagine, 0.3936; L-aspartate, 0.3936; L-cysteine, 0.1627; L-glutamine, 0.3881; L-glutamate, 0.3882; glycine, 1.7587; L-histidine, 0.1323; L-isoleucine, 0.4773; L-leucine, 0.741; L-lysine, 0.5021; L-methionine, 0.2218; L-phenylalanine, 0.2421; L-proline, 0.4147; L-serine, 0.4421; L-threonine, 0.4588; L-tryptophan, 0.0598; L-tyrosine, 0.1725; L-valine, 0.7785.

1.2.1. Justification for cell composition of Dehalobacter

To compare theoretical yields to qPCR-measured yields, the yield in gdw must be converted to yield in cells. This can be performed using measured cell sizes and assumptions of density. *Dehalobacter* cells are rod-shaped (3), thus the volume of an average *Dehalobacter* cell can be calculated as follows.

$$V_{cell} = \pi r^2 l = 0.31\ \mu m^3 \tag{Eq. 3}$$

where r = ½ the diameter (d) of one *Dehalobacter* cell, d = 0.3-0.5 μm (3)

l = length of one *Dehalobacter* cell = 2-3 μm (3)

Cell volume can be converted to wet mass using an estimated cell density of 1.03 g/mL (equal to water), and dry mass can be estimated by assuming a bacterial cell has 70% water as follows (4).

$$m_{cell} = 0.3(\rho_{H2O} V_{cell}) = 0.3(1.03 \times 10^{-12} \times 0.31) = 9.71 \times 10^{-14}\ \text{g/cell} \tag{Eq. 4}$$

where $\rho_{H2O} = 1.03 \times 10^{-12}$ ml/μm^3 (4).

$V_{cell} = 0.31\ \mu m^3$ (Eq. 3)

The percentage of DNA in 1 gdw can be calculated by first calculating the mass of a *Dehalobacter* genome.

$$m_{genome} = \frac{l_{genome} \times M_{bp}}{N_A} = \frac{(3.1 \times 10^6) \times 666}{6.023 \times 10^{23}} = 3.43 \times 10^{-15}\ \text{g} \tag{Eq. 5}$$

where l_{genome} = mean length of *Dehalobacter* genome = 3.1×10^6 bp

M_{bp} = an average molecular mass of one base pair = 666 g/mol,

N_A = Avogadro's number = 6.023×10^{23} molecules

So, the percentage of DNA in 1 gdw can be determined by:

$$\frac{m_{genome}}{m_{cell}} \times 100 = \frac{3.43 \times 10^{-15}}{9.71 \times 10^{-14}} \times 100 = 3.53\% \tag{Eq. 6}$$

where $m_{cell} = 9.71 \times 10^{-14}$ g/cell (Eq. 4)

No other experimental data relevant to biomass components were available for *Dehalobacter* and were thus estimated from the published *Methanosarcina barkeri* and *Bacillus subtilis* models, including proteins, lipids, carbohydrates, and soluble pools and ions used in this model (5, 6).

The cell composition has been previously shown to minimally affect growth predictions in metabolic modelling of many organisms, and growth predictions are typically robust to small changes in biomass composition (7–10). To confirm the minimal effects of biomass composition in strains SAD (**Figure S1A**) and DAD (**Figure S1B**), growth yields were compared within a range of biomass compositions (0.4-0.6 g of protein gdw^{-1}). Growth yields remained relatively stable between 0.45-0.55 g of protein gdw^{-1} (3-4% differences for strain DAD; 3-6% differences for strain SAD). These differences are insignificant compared to the differences between metabolic modes (20-80%) and compared to error in experimental growth data (20-30%).

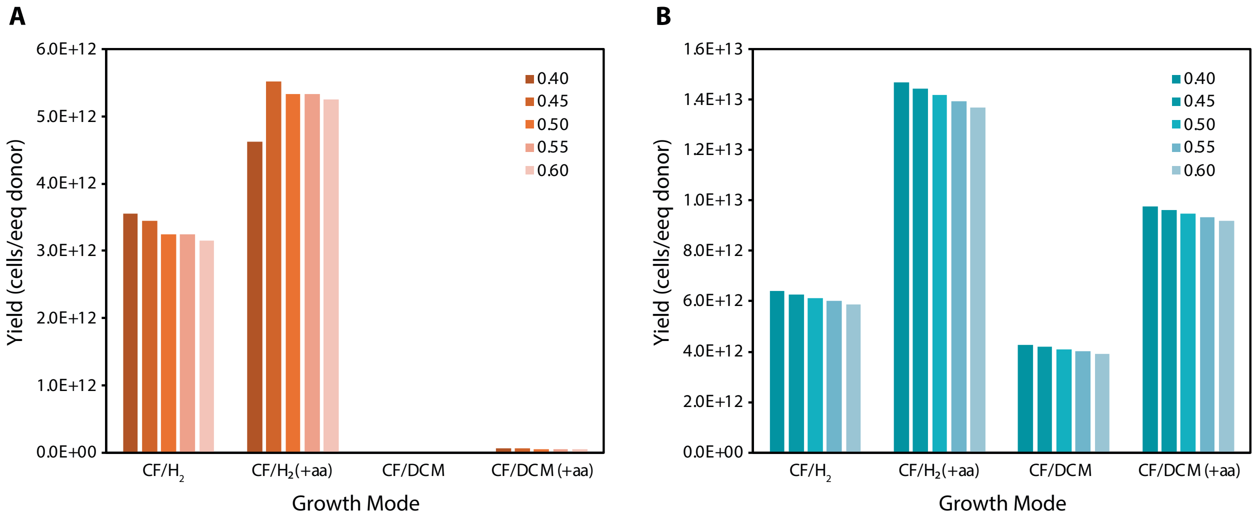

Figure S1. Impact of biomass composition on yield in *Dehalobacter* models **A**) iOB638 (strain SAD) and **B**) iOB649 (strain DAD). Biomass ranges reflect 0.4-0.6 g of protein gdw^{-1}.

1.3. Theoretical maximum energy transfer efficiency and proton translocation stoichiometry of *Dehalobacter* electron transport chain

The SC05 culture uses three electron donor/ acceptor pairs. The ΔG°′ of each reaction was calculated as follows, where the free energies of each acceptor and donor half reactions ($\Delta G°'_a$ and $\Delta G°'_d$) are calculated from the standard free energy of each compound (11), assuming standard conditions (1M concentration, 25°C, pH 7.0).

$$\Delta G^{\circ\prime}_{rxn} = \Delta G^{\circ\prime}_{a} - \Delta G^{\circ\prime}_{d} \quad \text{(Eq. 7)}$$

1.3.1. Theoretical maximum energy transfer efficiency (ATP/e⁻)

The theoretical maximum ATP/e^- ratio, $(n_{ATP}/n_{e-})_{\max}$ can be determined from the following equation, since $\Delta G^{\circ\prime} = -nF\Delta E^{\circ\prime}$ (12).

$$\left(\frac{n_{ATP}}{n_{e-}}\right)_{max} = \frac{\Delta E^{\circ\prime} F}{\Delta G'_P} = \frac{\Delta G^{\circ\prime}_{rxn}}{n\Delta G'_P}$$

(Eq. 8)

where F = Faraday constant = 96.5 kJ/ mol V

$\Delta E^{\circ\prime}$ = difference in standard redox potential between the electron donor and acceptor

$\Delta G'_P$ = free energy of phosphorylation under physiological conditions, pH 7

n = number of e^- transferred in the reaction (if $\Delta G^{\circ\prime}_{rxn}$ is on a per mole basis, n = 1)

The free energy of the phosphorylation reaction under physiological conditions ($\Delta G'$) can be calculated from the free energy of the phosphorylation reaction at standard conditions and pH 7 ($\Delta G^{\circ\prime}_P$) using:

$$\Delta G'_P = \Delta G^{\circ\prime}_P + RT \ln \frac{[\mathrm{ATP}]}{[\mathrm{ADP}][\mathrm{P}_i]} = 32 + (8.314 \times 298.15 \times \ln 1) = 49.12 \text{ kJ/mol}$$

(Eq. 9)

where $\Delta G^{\circ\prime}_P$ = 32 kJ/ mol (13)

R = 8.314 J/ mol K (universal gas constant)

T = 298.15 K (absolute temperature at 25°C)

assuming [ATP] = [ADP], $[P_i]$ = 1 mM

To calculate $\Delta G^{\circ\prime}_{rxn}$, the $\Delta G^{\circ\prime}$ of the electron donor half reaction is added to that of the acceptor half reaction. The $\Delta G^{\circ\prime}$ of each donor and acceptor half reaction can be calculated by combination of the standard free energies of formation of each compound in the half reaction (listed in **Table S1**), multiplied by its stoichiometry on an eeq basis. All relevant half reactions are summarized in **Table S2**.

Table S1. Standard free energies of formation (1 M concentration, 25°C, pH 7).

**Compound**	**ΔG°′** (kJ/mol)	**Reference**
CO_2 (g)	–394.38	(14)
HCO_3^- (aq)	–586.85	(14)
H_2O (l)	–237.19	(14)
H^+ (aq, pH = 7)	–39.87	(14)
H_2 (g)	0.00	(14)
CF (aq)	–66.50	(15)
DCM (aq)	–66.11	(15)
Cl^- (aq)	–131.20	(13)

Table S2. Half reactions (as reductions) and their Gibb's standard free energy (unit activity 25°C, pH 7).

**Compound**	**Half reaction**	**eeq/ mole**	**ΔG°′** (kJ/ eeq)
DCM	$CO_2 + 1/2\ Cl^- + 3/2\ H^+ + e^- \rightarrow 1/4\ CH_2Cl_2 + 1/2\ H_2O$	4	88.88
CF (to DCM)	$1/2\ CHCl_3 + H^+ + e^- \rightarrow 1/2\ CH_2Cl_2 + 1/2\ H^+ + 1/2\ Cl^-$	2	–45.47
Hydrogen	$H^+ + e^- \rightarrow 1/2\ H_2$	2	39.87
Methane	$1/8\ CO_2 + H^+ + e^- \rightarrow 1/8\ CH_4 + 1/4\ H_2O$	8	23.52
Cell synthesis	$1/5\ CO_2 + 1/20\ NH_4^+ + 1/20\ HCO_3^- + H^+ + e^- \rightarrow 1/20\ C_5H_7O_2N + 9/20\ H_2O$	20	NA

All $\Delta G^{\circ\prime}_{rxn}$ can be converted to $\Delta G'_{rxn}$ values reflecting experimental conditions (still at pH 7) using the following equation:

$$\Delta G'_{rxn} = \Delta G^{\circ\prime}_{rxn} + RT \ln \frac{[\text{products}]}{[\text{reactants}]}$$

(Eq. 10)

where R = 8.314 J/ mol K (universal gas constant)

T = 298.15 K (absolute temperature at 25°C)

[CF] = 0.001 M

[DCM] = 0.001 M

$[Cl^-]$ = 0.033 M

$[CO_2]$ = 0.2 atm

$[H^+] = 1\times 10^{-7}$ M (pH 7)

$[H_2] = 1\times 10^{-4}$ atm

To calculate theoretical maximum ATP/e^-, during dechlorination of CF to DCM for example, (Eq. 8 is used as follows.

$$\left(\frac{n_{ATP}}{n_{e-}}\right)_{max} = \frac{\Delta G'_{rxn}}{n\Delta G'_P} = \frac{-85}{1 \times 49.12} = 1.73$$

(Eq. 11)

where $\Delta G'_{rxn}$ = –97.8 kJ/ eeq (Eq.10)
$\Delta G'_P$ = –49.12 kJ/ mol (Eq. 9)
n = number of e^- transferred in the reaction ($\Delta G^{\circ\prime}_{rxn}$ is on a per mole basis, so n = 1)

These calculations were performed for each ΔG_{rxn}, as shown in **Table S3**.

Table S3. Selection of theoretical thermodynamic constraints for energy metabolism of *Dehalobacter*.

**Mode** (donor/ acceptor)	$\Delta G^{o\prime}_{rxn}$ (kJ/eeq)	ΔG_{rxn}	**ATP/*e*⁻** (theoretical maximum)	**H^+ transloc.** (/mol ATP)	**H^+/e^- ratio** (max)	(assumed)	**Efficiency** (assumed/ max)	**ATP/*e*⁻** (assumed)	(expt'l)*
						8	1.01	1.99	
						7	0.88	1.75	
						6	0.75	1.50	
1	–85	–97.8	1.99	4	7.96	5	0.63	1.25	0.5
						4	0.50	1.00	
						3	0.38	0.75	
						2	0.25	0.50	
						1	0.13	0.25	
						11	0.99	2.75	
						10	0.90	2.50	
						9	0.81	2.25	
						8	0.72	2.00	
						7	0.63	1.75	
2	–134	–137.1	2.79	4	11.16	6	0.54	1.50	NA
						5	0.45	1.25	
						4	0.36	1.00	
						3	0.27	0.75	
						2	0.18	0.50	
						1	0.09	0.25	
						7	1.07	1.75	
						6	0.91	1.50	
						5	0.76	1.25	
3	–49	–80.6	1.64	4	6.56	4	0.61	1.00	NA
						3	0.46	0.75	
						2	0.30	0.50	
						1	0.15	0.25	

*Reference (16)

1.3.2. Proton translocation stoichiometry (H^+/e^- ratio)

Assuming 4 protons are translocated across the cell membrane during ADP phosphorylation (17), we obtain the theoretical maximum H^+/e^- during CF dechlorination using the theoretical maximum ATP/e^- as follows.

$$\left(\frac{n_{H^+}}{n_{e-}}\right)_{max} = 4\left(\frac{n_{ATP}}{n_{e-}}\right)_{max} = 4(1.73) = 6.92$$

(Eq. 12)

If the theoretical maximum H^+/e^- of dechlorination process is 6.92, the H^+/e^- should be either 6 or 7. A lower H^+/e^- ratio of 1.25 ± 0.2 has been measured experimentally for H_2-dependent reductive dechlorination of tetrachloroethene by *Dehalobacter restrictus* PER-K23 (16). Based on these experiments, Schumacher and Holliger suggested a model of vectorial proton transport for dechlorination, resulting in a theoretical H^+/e^- ratio of 1.5 (16). Calculations by Adrian and Löffler aligned published growth yields with this data, wherein measured *Dehalobacter* yields of 1.6–2.8 gdw/e^- equate to 0.25–0.5 mol ATP/e^-,

assuming a biomass yield of 5–10 gdw mol^{-1} ATP (18). The assumed ATP/e^- value (0.50) corresponding to 1 H^+/e^- (**Table S3**) agreed with the maximum experimental ATP/e^- value of 0.5 mol ATP/e^- (18), and the proton translocation stoichiometry of CF dechlorination (via menaquinone oxidation) in *Dehalobacter* was chosen as 2 H^+/e^-.

1.4. Calculation of maintenance parameters

ATP is required for cellular maintenance (i.e. macromolecule polymerization, amino acid turnover, ion transport). This maintenance energy can be subdivided into growth associated maintenance (GAM, related to cell division) and non-growth associated maintenance (NGAM, related to homeostasis). Both GAM and NGAM can be calculated from chemostat data during growth of an isolate culture, but when lacking this data, NGAM can be calculated from the decay rate of the organism of interest (Eq. 12).

$$\text{NGAM} = \frac{b}{Y_G} = \frac{b}{Y} \qquad \text{(Eq. 13)}$$

where b = specific maintenance rate or decay rate (d^{-1})
Y_G = True growth yield/ yield without maintenance (gdw/eeq)
$Y_G = Y$ = Observed growth yield

In *Dehalobacter* strains SAD and DAD, GAM was ultimately set at 60 mmol ATP gdw^{-1}, reflecting experimental values measured in *Dehalococcoides mccartyii*—another dechlorinating microbe (19). Though *Dehalobacter* has a higher growth rate than *Dehalococcoides* (1.6–2.8 gdw/eeq (18)), it has a lower decay rate (0.017 d^{-1}). This mirrors the low decay rate of other anaerobes in bioremediation cultures, like ORM2, which has a decay rate of ~0.01 d^{-1} (20). Using these parameters for *Dehalobacter*:

$$\text{NGAM} = \frac{b}{Y} = \frac{0.017 \times 2 \times 1000}{2.2 \times 4} = 3.86 \text{ mmol ATP gdw}^{-1}\text{d}^{-1} \qquad \text{(Eq. 14)}$$

where b = 0.017 d^{-1} (Shuping Wang, personal communication)
Y = 2.2 gdw/eeq donor (mean reported in ref (18))
assuming a H^+/ATP ratio of 4 (17)
assuming a H^+/e^- ratio of 2 (**Table S3**)

1.5. Validation of thermodynamic constraints compared to experimental data

The experimentally-determined H^+/e^- ratio of 1.25 ± 0.2 (16) is consistent with the H^+/e^- ratio predicted in Modes 1 and 2 (Table 3). Published biomass yields [5–10 gdw/mol ATP (18)] are in agreement with the FBA predictions (Table 3), but an order of magnitude lower than the thermodynamic max, which reinforces that mechanistic or thermodynamic restrictions also hinder complete exploitation of the free energy available in dechlorination reactions even without DCM mineralization (16).

2. Genomic curation

This section summarizes the comparative genomics between *Dehalobacter* strains SAD and DAD, as pertaining to energy metabolism, dechlorination, and carbon metabolism. All added or edited reactions are summarized in **Table S4**, according to the localization and expression described in **Table S5**. Genome alignments demonstrating presence and absence of differentially-encoded metabolic genes are shown in **Figure S2**. Proteins identified by proteomics are shown in **Table S8** in the accompanying excel.

Table S4. Reactions added to each *Dehalobacter* metabolic model based on genome presence. NA: not applicable (gene-free reaction), ND: not detected.

			Locus tag	
**Reaction**	**Name**	**Description**	**SAD** (WHF41_)	**DAD** (WHF31_)
RDase0	CF-dechlorinating RDase	cf_p + mql8_i --> cl_p + dcm_p + h_p + mqn8_i	12130-45	05425-35
MecEBF	DCM assimilation	dcm_c + thf_c --> 2.0 cl_c + 2.0 h_c + mlthf_c	12045-95	05465-5510
DCMtpp	DCM diffusion	dcm_p <=> dcm_c	NA	NA
DCMtex	DCM transport	dcm_p <=> dcm_e	NA	NA
CFtex	CF transport	cf_e --> cf_p	NA	NA
Cltd	HCl transport	cl_c + h_c <=> cl_e + h_e	12045	05510
EX_cf_e	Exchange of CF	cf_e <--	NA	NA
EX_dcm_e	Exchange of DCM	dcm_e <=>	NA	NA
FTHFD	Formyltetrahydrofolate deformylase	10fthf_c + h2o_c <=> for_c + h_c + thf_c	00805	01360
CODH_ACS	CODH/Acetyl-CoA synthase (Wood-Ljungdahl)	co2_c + coa_c + 2.0 fdxrd_c + h_c + mecfsp_c <=> accoa_c + cfesp_c + 2.0 fdxox_c + h2o_c	00745-75	01305-35
HYD-NADH	Hydrogen:NAD+ oxidoreductase	h2_c≈ + nad_c <=> h_c + nadh_c	13630,13900	03885, 03645
STN	*Sporomusa*-type Nfn	fdxox_c + nad_c + 2.0 nadph_c <=> fdxrd_c + h_c + nadh_c + 2.0 nadp_c	09315-25	08170-80
FDXMQpp	fdx:mql oxidoreductase	2.0 fdxrd_c + 6.0 h_c + mqn8_i –-> 2.0 fdxox_c + 4.0 h_p + mql8_i	4980-5025	12570-620
ICDHyr	Isocitrate dehydrogenase	icit_c + nadp_c --> akg_c + co2_c + nadph_c	10130	07365
FRD2	Fumarate reductase	fum_c + mql8_i <=> mqn8_i + succ_c	ND	11480
NIT1b	Nitrogenase (ATP-hydrolysing)	16.0 atp_c + 8.0 fdxrd_c + 16.0 h2o_c + n2_c <=> 16.0 adp_c + 8.0 fdxox_c + h2_c + 6.0 h_c + 2.0 nh4_c + 16.0 pi_c	ND	01830-85
ASPT	Aspartate ammonia-lyase	asp__L_c --> fum_c + nh4_c	ND	02985

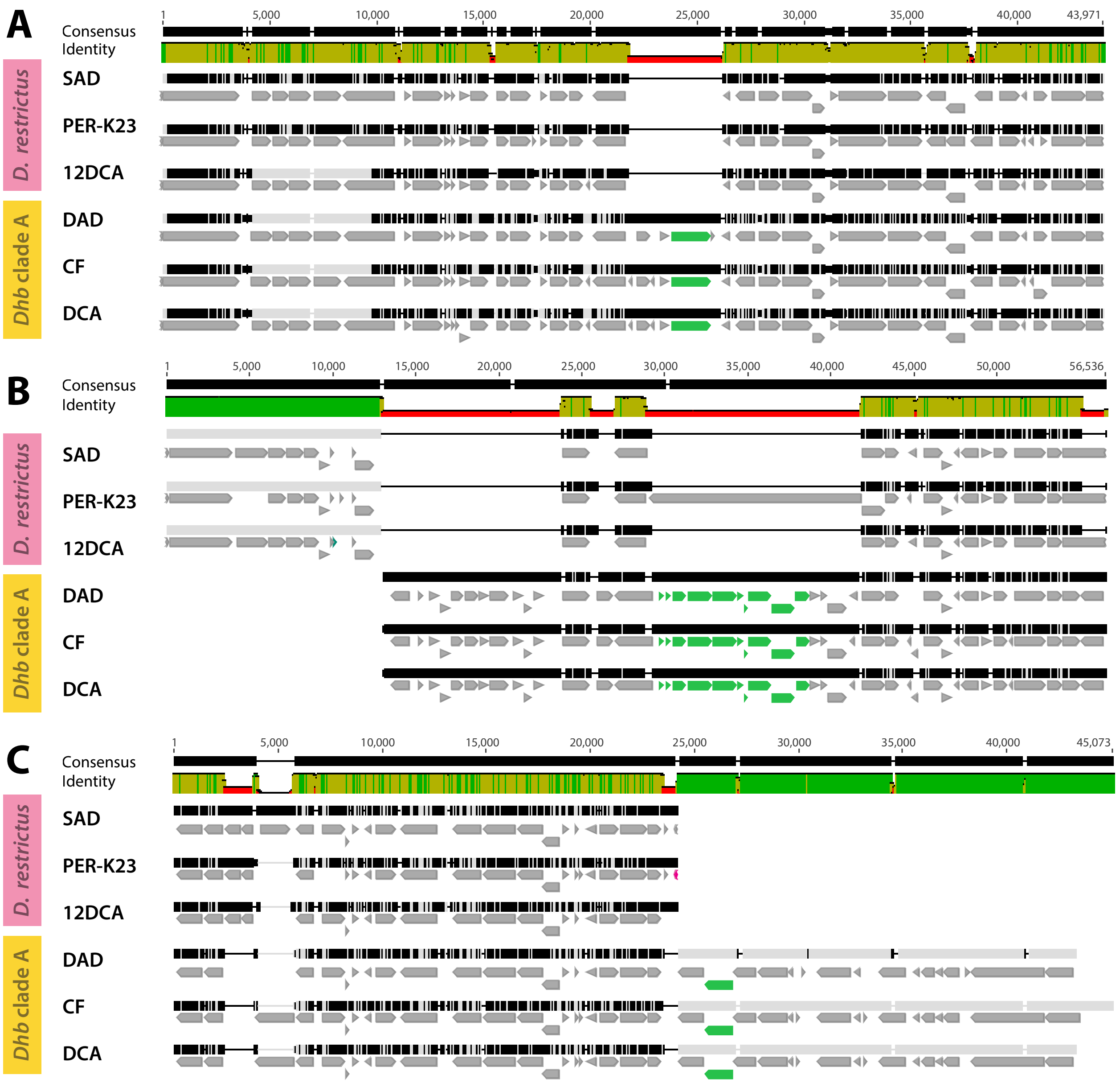

Figure S2. Alignment of key metabolic gene differences in *Dehalobacter* strains SAD and DAD, as well as other closed genomes in each *Dehalobacter* clade (DAD: Clade A, SAD: *Dehalobacter restrictus* = Clade B). Key genes are highlighted in green, including A) fumarate reductase, B) nitrogenase gene cassette, and C) aspartate ammonia-lyase.

Table S5. Genomic localization and proteomic expression of key metabolic reactions in *Dehalobacter* strains SAD and DAD. Reaction ID (with brackets) = multiple reaction IDs to test NAD(P)H promiscuity. Proteins measured during Mode 2 (CF dechlorination) in SC05-UT, and Mode 3 (DCM mineralization) in DCME are marked with the mode they were detected in. ND: not detected. Italicization: homologous proteins are too similar for strain-specific resolution.

					SAD		DAD	
	**Reaction ID**	**Reaction**	**Description**	**Gene**	**Locus tag** (WHF41_)	**Mode**	**Locus tag** (WHF31_)	**Mode**
Dehalo-genation	RDHase0	cf_p + mql8_i --> cl_p + dcm_p + h_p + mqn8_i	Reductive dehalogenase, catalytic subunit	RdhABC	12130-45	2	05425-35	3
	MecEBF	dcm_c + thf_c --> 2.0 cl_c + 2.0 h_c + mlthf_c	Histidine kinase and PocR sensor	Mec cassette	12045-95	2	05465-5510	3
Hydrogenases	H2CYTb	cytbdox_i + h2_p --> cytbdred_i + 2.0 h_p	[NiFe] Group 1a: H2-uptake (unidirectional)	HYD-1	05830	ND	11455	ND
				HYD-8	03090	ND	02665	3
			[NiFe] Group 1d: H2-uptake (unidirectional)	HYD-7	14080	ND	03470	ND
	HYDA_ech	2.0 fdxrd_c + 4.0 h_c --> 2.0 fdxox_c + h2_c + 2.0 h_p	[NiFe] Group 4e: Ech-type, Bidirectional	HYD-2	08860	2,3	08645	2,3
	HYDFDN (2)	2.0 fdxox_c + 2.0 h2_c + nad(p)_c --> 2.0 fdxrd_c + 3.0 h_c + nad(p)h_c	[FeFe] Group A3: Electron-bifurcation, Reversible	HYD-3	09315	2,3	08180	2,3
	HYD-NADH	h2_c + nad_c <-- h_c + nadh_c	[FeFe] Group A3: NADH-oxidizing hydrogenase	HYD-4	13630	ND	03885	ND
				HYD-6	13900	2	03645	2
WLP	FDH(6r)	for_c + nad(p)_c --> co2_c + nad(p)h_c	Formate dehydrogenase	FdhAEFH	13770-80	ND	03760-70	ND
	FHL	for_c + h_c --> co2_c + h2_c	Formate hydrogenolyase	EhfABCDEF (FdhF)	8475-505	ND	08985-9010	ND
	FDH5pp	for_p + h_p + mqn8_i --> co2_p + mql8_i	Formate dehydrogenase-N	FdoGHI	09275-90	2	08205-20	ND
	MTHFD (2)	mlthf_c + nad(p)_c --> methf_c + nad(p)h_c	Methylene-THF dehydrogenase		00795	*2,3*	01350	*2,3*
	MTHFC	10fthf_c + h_c <=> h2o_c + methf_c	Methenyl-THF cyclohydrolase		00800	2	01355	3
	FTHFLi	atp_c + for_c + thf_c --> 10fthf_c + adp_c + pi_c	Formate-THF ligase	FTHFD	00805	ND	01360	*2,3*
	CODH_ACS	co2_c + coa_c + 2.0 fdxrd_c + h_c + mecfsp_c <=> accoa_c + cfesp_c + 2.0 fdxox_c + h2o_c	CODH/Acetyl-CoA synthase (Wood-Ljundahl)	AcsAB	00745/75	2	01305/35	3
	METR	5mthf_c + cfesp_c <=> mecfsp_c + thf_c	5-methyl-THF corrinoid/ FeS protein methyltransferase	AcsE	00750/90	*2,3*	01310/45	*2,3*
	ACS	ac_c + atp_c + coa_c <=> accoa_c + amp_c + ppi_c	Acetyl-CoA synthetase		06690	2	10600	ND
	MTHFR (2_1, 3_1)	5mthf_c + nad(p)_c <-- h_c + mlthf_c + nad(p)h_c	Methylene-THF reductase		00815	2	01370	3

Table S5. *Continued.*

	Reaction ID	Reaction	Description	Gene	SAD		DAD	
					**Locus tag** (WHF41_)	**Mode**	**Locus tag** (WHF31_)	**Mode**
Energy	FDXMQpp	2.0 fdxrd_c + 6.0 h_c + mqn8_i --> 2.0 fdxox_c + 4.0 h_p + mql8_i	Ferredoxin:menaquinol oxidoreductase (Complex 1)	NDH	4980-5025	ND	12570-620	ND
	NFNAB	2.0 fdxrd_c + h_c + nadh_c + 2.0 nadp_c --> 2.0 fdxox_c + nad_c + 2.0 nadph_c	electron-bifurcating transhydrogenase	nfn	07060-65	ND	10230-35	ND
	STN		Sporomusa-type Nfn	stnEFG	09315-25	*2,3*	08170-80	*2,3*
	ATPS4rpp	adp_c + 4.0 h_p + pi_c <=> atp_c + h2o_c + 3.0 h_c	ATP synthase	ATPs	16390-430	2,3	15675-715	3
TCA/ carbon	ME(1, 2)	mal__L_c + nad(p)_c --> co2_c + nad(p)h_c + pyr_c	malic active enzyme	MAE	06535	2	10770	ND
	FUM	mal__L_c <=> fum_c + h2o_c	fumarate hydratase	FH	15250-55	ND	14345-50	ND
	FRD2	fum_c + mql8_i ⇌ mqn8_i + succ_c	succinate dehydrogenase/ fumarate reductase	SDH	NF	ND	11480	ND
	SUCOAS	atp_c + coa_c + succ_c <=> adp_c + pi_c + succoa_c	succinate coA ligase	SCL	06515-25	2	10780-90	ND
	OORr	akg_c + coa_c + 2.0 fdxox_c --> co2_c + 2.0 fdxrd_c + h_c + succoa_c	2-oxoglutarate/2-oxoacid ferredoxin oxidoreductase subunit alpha/beta	korAB	09510-20	ND	07935-45	ND
	GLUDy	akg_c + h_c + nadph_c + nh4_c --> glu__L_c + h2o_c + nadp_c	glutamate dehydrogenase	GDH	13925-35	*2,3*	03605-15	3
	ICDHyr	icit_c + nadp_c --> akg_c + co2_c + nadph_c	isocitrate dehydrogenase	ICDH	10130	ND	07365	ND
	ACONTa	cit_c <=> acon_C_c + h2o_c	aconitate hydratase	ACO	09410-15	ND	08040-45	ND
	ACONTb	icit_c <=> acon_C_c + h2o_c						
	CITL	cit_c <=> ac_c + oaa_c	citrate synthase	CS	15540; 16030	*2*	15095; 14640	*2*
	POR_syn	accoa_c + co2_c + 2.0 fdxrd_c + h_c --> coa_c + 2.0 fdxox_c + pyr_c	Pyruvate ferredoxin/flavodoxin oxidoreductase	POR	15695	ND	14775	ND
					0680	2,3	01230	ND
					NF	ND	01010	ND

2.1. Curation of energy metabolism

2.1.1. Dechlorination and electron transport

Though the genomes of *Dehalobacter* strains SAD and DAD encode 27 and 17 RDases, respectively, expression of only one highly similar RDase from each strain has been detected (21). The expressed RDase from strain SAD, AcdA, was experimentally determined to be most active using chloroform as an electron acceptor, which it reduces to DCM (21). The RDase from strain DAD (dubbed "BcdA" has not been purified, but shares >99% identity to AcdA (21, 22). Thus, the electron acceptor reaction in both models was adjusted accordingly and constrained based on experimental uptake rates (~10 mmol gdw^{-1} d^{-1}). Reduction of CF is mediated through cytochrome b and menaquinone, using electrons from H_2, a known electron donor for *Dehalobacter*. However, genomic presence of an 11-subunit Complex I-like gene cluster suggests the existence of alternative entry points into the electron transport chain (ETC). This cluster is lacking the NADH-oxidizing module of Complex I, and has been suggested to act as a dock for various electron donors, such as ferredoxins, in a related genus—*Desulfitobacterium*—when using complex electron donors rather than hydrogen (23).

2.1.2. Hydrogenase classification

The two *Dehalobacter* genomes encode 8 homologous putative hydrogenases—labelled Hyd-1 through Hyd-8—which were classified using HYDdb (**Table S6**, **Figure S3**) (24). These included 3 [FeFe] electron-bifurcating hydrogenases, 3 [NiFe] Hup-type periplasmic uptake hydrogenases, and one [NiFe] formate-coupled respiratory hydrogenase. Though the latter was previously annotated as a Hyf-type or Hyc-type formate hydrogenlyase (FHL, [NiFe] Group 4a), it more closely resembles a divergent 6-subunit hydrogenase encoded by several classes of Firmicutes from anoxic soils ([NiFe] Group 4f) (24, 25). Its activity has not been confirmed biochemically, and despite the prediction that it may couple its activity to proton translocation using its antiporter-like domains, in this work we assume its metabolic function is similar to a prototypical FHL.

One homologous energy-conserving hydrogenase (Ech-type) was detected in both strains, but strain DAD included one additional Ech-type hydrogenase (Hyd-9; **Table S6**, **Figure S3**). This hydrogenase appears to be species-specific; Hyd-9 was not present in strain SAD or strain PER-K23 (which are both strains of *Dehalobacter restrictus*) but is present in other *Candidatus* Dehalobacter alkaniphilus strains (strains CF and DCA). This additional Ech-type copy may increase the ability to highly express this hydrogenase, but further work is required to understand these implications.

A reaction for each category of hydrogenase was amended to the model and constrained for directionality. Two SAD hydrogenases and three DAD hydrogenases matched to the novel proteomic

database presented in this study (**Table S6**, shaded blue), and two SAD hydrogenases and one DAD hydrogenase (**Table S6**, shaded purple) were found in a previous study (21, 26), thus were constrained appropriately in the models.

Table S6. Classification of hydrogenases in each *Dehalobacter* strain, and cofactors encoded downstream of the catalytic gene and predicted by HYDdb. Highlighted locus tags were found expressed via proteomics.

		**Locus tags** (catalytic subunit)							**Cofactors**	
**ID**	**PER-K23** (Dehre_)*	**SAD** (WHF41_)	**DAD** (WHF31_)	**AA %ID**	**Group**	**SU**	**Activity**	**Localization**	**Adjacent gene**	**HYDdb**
Hyd-1	01404 (1061)	05830	11455	92.5	[NiFe] Group 1a	3	Uptake	Periplasm	cytb	cyt c3
**Hyd-2**	**08335** (1645)	**08860**	**08645**	95.3	[NiFe] Group 4e	6	Ech-type	Membrane-bound	fdx	fdx
**Hyd-3**	**08805 (1739)**	**09315**	**08180**	99.5	[FeFe] Group A3	3	Bifurcation, reversible	Cytosol	fdx	NAD, fdx
Hyd-4	11735 (2317)	13630	03885	99.2	[FeFe] Group A3	4	Bifurcation, reversible	Cytosol	fdx fdx	NAD, fdx
**Hyd-5**	**07945 (1569)**	08480	09005		[NiFe] Group 4f	6	Formate-coupled	Membrane-bound	None	None
**Hyd-6**	**12025** (2374)	**13900**	**03645**	99.5	[FeFe] Group A3	3	Bifurcation, reversible	Cytosol	fdx	NAD, fdx
Hyd-7	12185 (2405)	14080	03470	100.0	[NiFe] Group 1d	3	Uptake	Membrane-bound	cytb	cytb, quinone
**Hyd-8**	**02790 (0551)**	**03090**	**02665**	97.7	[NiFe] Group 1a	3	Uptake	Periplasm	cytb	cyt c3
Hyd-9	NA	NA	11965	NA	[NiFe] Group 4e	6	Ech-type	Membrane-bound	fdx	fdx

*Accession numbers for strain PER-K23 are reported from the annotations in NCBI genome CP007033, as well as from a previous annotation version (in brackets) referenced in Rupakula *et al.* (27) for easier cross referencing.

One of these expressed hydrogenases (**Table S6**, Hyd-3, [FeFe] Group A3) was previously identified as a homolog of the *Sporomusa*-type NfnAB electron-bifurcating oxidoreductase (28). Previous work has suggested that an electron bifurcating transhydrogenase (NfnAB-type) is present in *Dehalobacter* genome, but its activity has not been characterized (1). Its dominant activity in *Moorella thermoacetica* is the reduction of NADP via reduced ferredoxin (fdx_{red}) and NADH, whereas STN has been shown to reduce ferredoxin (fdx_{ox}) and NAD using NADPH (28, 29). Both genomes encode many uncharacterized oxidoreductases, thus NAD and NADP-dependent ferredoxin reductases were assumed to be in the model.

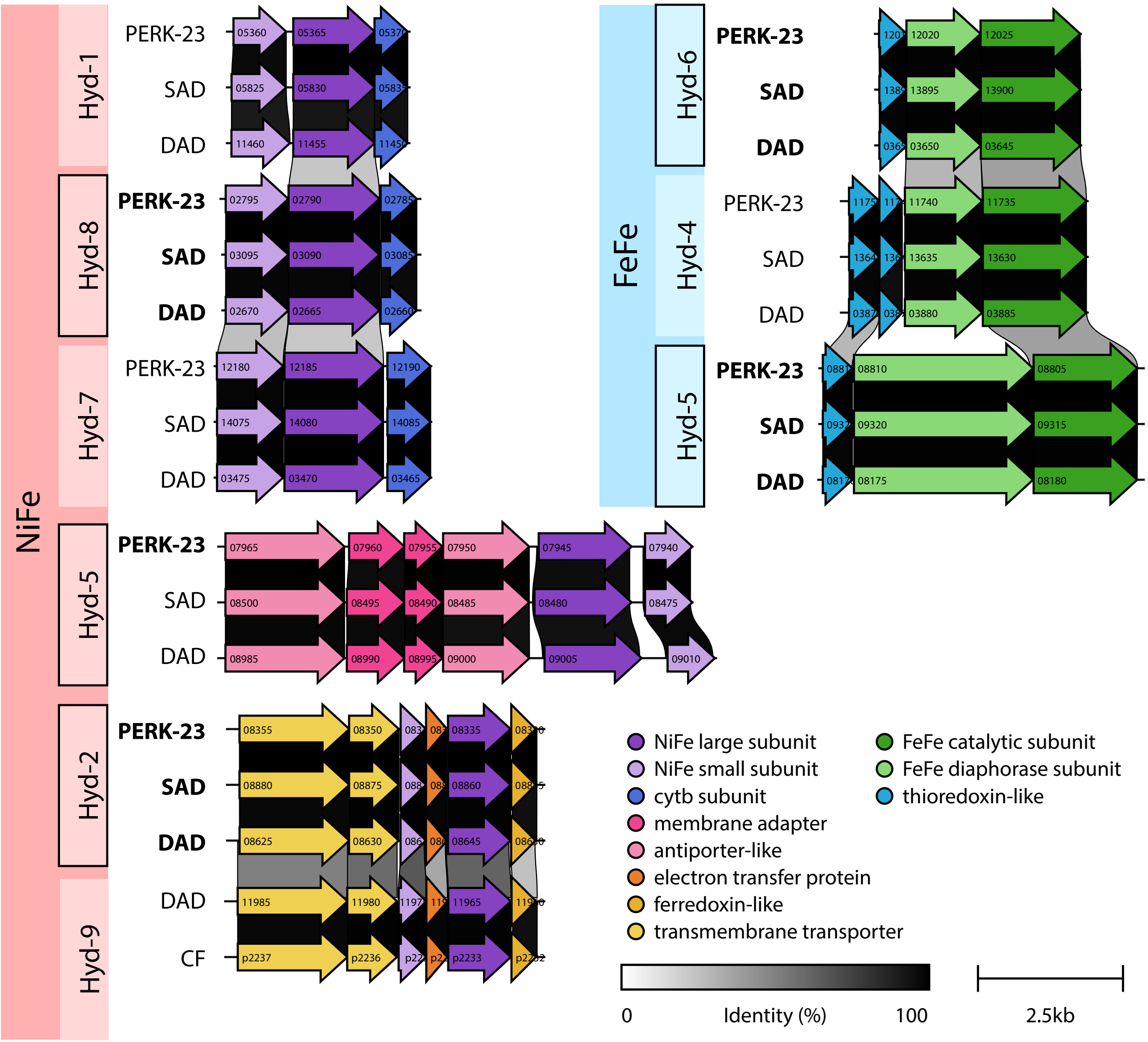

Figure S3. Alignment of hydrogenase gene clusters in in *Dehalobacter* strains SAD and DAD and their predicted classifications, compared to strain PER-K23 (and strain CF, in the case of Hyd-9). Subunits are colored by function and linked by percent identity, as clustered using clinker (30). Locus tags label each gene and are abbreviated for each strain (genome accession in brackets): SAD (CP148032), "WHF41_"; DAD (CP148031), "WHF31_"; PER-K23 (CP007033), "Dehre_"; CF (CP003870), "DCF50_".

2.1.3. *Formate metabolism*

Three formate dehydrogenases were also encoded by each genome, as previously found in other strains of *Dehalobacter*: NAD(P)-dependent formate dehydrogenase, menaquinone-based formate dehydrogenase, and formate-hydrogen lyase (**Table S5**)—all of which require molybdopterin for activity. The traditional molybdopterin biosynthesis pathway is incomplete in both strains; they each lack two subunits of molybdopterin synthase: *moaD* and *moaE.* This trait is common to all known *Dehalobacter* strains, but in strain SAD, like strains PER-K23 and E1, two MOSC domain-containing proteins

(SC05P_02627 and SC05P_02674) were identified in the same gene cluster as the other molybdopterin biosynthesis enzymes. The MOSC domain is predicted to mediate the formation of diverse sulfur-metal clusters and has been proposed as a potential substitute for molybdopterin synthase in *Dehalobacter* (31, 32). Strain DAD, like strains CF and DCA, only possesses one MOSC domain-containing protein (DCME_00761). If this single protein is insufficient for molybdopterin biosynthesis, these strains may possess an alternative non-conventional enzyme, or they may receive molybdopterin from another microbe in the community.

Table S7. Summary of key genera encoding genes molybdopterin biosynthesis PFAM/TIGRFAM motifs in the SC05-UT metagenome. Copies of each gene by HMM are highlighted in green, and missing steps are highlighted in orange.

		**Archaeal genera**			**Bacterial genera**															
		Methanoregula	*Methanothrix*	*Methanomethylovorans*	*Bellilinea*	*Pseudomonas*	Delta-02	*Levilinea*	UBA2286	UBA467	QAXR01	UBA11384	PHBD01	UBA5368	Syner-01	T78	UBA4066	*Desulfovibrio*	*Dehalobacter*
moaA	TIGR02666				2	2	1	3	2		1		1	1	2	1	2	1	1
	TIGR02668	2		1						1		2							
moaB	TIGR00176	2	1	1			1		2	1	1	2	1	1					1
	TIGR00177	1	4	1	5	2	2	4	4	6	4	2	1	2	1	5	3	1	3
	TIGR02667					1													
moaC	TIGR00581	2	2		1	1	2	2	2	1	1	1	1	1	2	1	2	1	2
moaD	TIGR01682				1														
	TIGR01687	5	4	1	2			1							5		2		
moaE	PF02391	3	2	2	1	1	2	1	2	1	1	3	1	1		1		1	
mobA	PF12804	17	9	5	4	7	15	16	14	9	7	4	11	5	19	16	1	9	6
	TIGR02665					2													
moeA	PF03453	9	4	3	3	1	4	4	2	4	3	3	1	1	4	5	4	2	1
moeB	PF00899	4	1	1	6	2	7	1	1	1	4	3	3	1	2	3	2	2	1
mogA	PF00994	14	5	3	5	2	8	8	6	6	7	4	3	2	13	1	8	5	4
	total HMMS	10	9	9	10	10	9	9	9	9	9	9	9	9	8	8	8	8	8

Other microbes in SC05 are capable of molybdopterin biosynthesis, and nutrient cross feeding has been previously identified in *Dehalobacter*-containing enrichment cultures (33, 34). Partial putative molybdopterin biosynthesis pathways were found assigned to many (458 genera and 288 families) in the SC05-UT metagenome due to spurious similarity to other enzymes. All hits and their predicted taxonomies

are shown in **Dataset 1 (**DOI:10.6084/m9.figshare.26972683**).** Only taxa with more than seven of the nine biosynthetic genes were summarized in **Table S7**. Three archaeal genera and four bacterial genera encode all biosynthesis steps, but closure and further analysis of these genomes is necessary for confirming their metabolic capacity and further analyzing these potential exchanges. Nonetheless, we have opted to allow these reactions to function, as their enzymes are expressed by *Dehalobacter* (27).

2.2. DCM metabolism and the Wood-Ljungdahl pathway

DCM has been experimentally determined to assimilate to methylene-THF in other anaerobic DCM-degraders (35, 36). More recently, the *mec* cassette was proposed to perform this reaction using a pair of methyltransferases and a methyl-binding corrinoid protein (37, 38). These enzymes have not been biochemically characterized; the precise mechanism and occurrence of intermediates remains unclear. Due to lack of a clear mechanism, DCM assimilation to methylene-THF was added as a single lumped reaction (MecEBF, **Table S4**). Methylene-THF metabolism occurs through the Wood-Ljungdahl pathway (WLP). Each strain's genome encoded a complete WLP (**Table S5**), and formyltetrahydrofolate deformylase and CO dehydrogenase/ acetyl-CoA synthase were added to the models accordingly (**Table S4**). The *mec* cassette also encodes a predicted chloride transporter (MecJ), which was added to the model (Cltd).

2.3. TCA cycle and amino acid metabolism

Dehalobacter is known to require amino acid supplementation when grown in isolation (27, 33, 39), due in part to its fragmented TCA cycle. Lack of malate dehydrogenase has been predicted to cause NADH/ NADPH redox imbalances within the metabolism despite its "rescue" with NADP-dependent malic active enzyme (33). Fumarate reductase (aka succinate dehydrogenase) is also reportedly lacking in *Dehalobacter* (31, 33, 39). No malate dehydrogenase was found in the genome of either strain SAD or DAD, but the catalytic subunit of fumarate hydratase was found in strain DAD (**Table S5**), thus was added to the DAD model *i*OB649 (**Table S4**). This differential possession of fumarate dehydratase, along with aspartate ammonia lyase and a nitrogen fixation cassette, were the only genomic differences found with respect to the key modules of the *Dehalobacter* strains' metabolisms, though extra copies of several amino acid biosynthesis genes, as well as Complex I, are present in strain DAD.

2.4. Vitamins and cofactors

Dehalobacter spp. can biosynthesize all essential cofactors, besides thiamin, biotin, and lipoic acid (3, 31). The isolated *D. restrictus* PER-K23 requires thiamin supplementation for growth, but does not require biotin (3). In SC05-UT and DCME, vitamin exchanges may occur between organisms, but are also provided in the mineral media used for growth (**Table S8**). Therefore, no reactions whose enzymes require these cofactors were inhibited in the FBA simulations.

Table S8. Cofactor and vitamin biosynthesis pathways in each species, and supplementation in mineral medium; Y: present, N: absent.

	D. restrictus	***Ca.* D. alkaniphilus**	**Mineral medium**
**Riboflavin (FMN, FAD)**	Y	Y	Y
**Nicotinate (NAD)**	Y	Y	Y
**Terpenoid**	Y	Y	
**Menaquinone**	Y	Y	
**Ferredoxins**	Y	Y	
**Folate**	Y	Y	Y (folate + PABA)
**CoA**	Y	Y	Y (pantothenic acid)
**Cobalamin (B_{12})**	Y	Y	Y
**Heme**	Y	Y	
**Pyridoxal phosphate PLP (B_6)**	Y	Y	Y
**Thiamin (B_1)**	partial	partial	Y
**Molybdopterin**	Y	Y	
**Lipoic Acid**	partial	N	Y
**Biotin (B_7)**	N	N	Y
**Coenzyme M**	NA	NA	Y

3. Flux balance analysis in *Dehalobacter* strains SAD and DAD

This section describes solutions to various modes and conditions under which FBA was performed. A description (**Table S9**) and summary (**Table S11)** of each simulation case can be found to accompany the full flux distribution (**Table S10**), all in the accompanying excel. Figures representing these flux distributions are shown below.

3.1. Abbreviation key (reactions and metabolites)

For brevity, metabolite and enzyme codes used in **Figures S1-S5** are listed below:

**Code**	**Reaction**
ACONT	aconitrate hydratase
ACS	Acetate-CoA ligase
ALATA	alanine:oxoglutarate aminotransferase
ATPS	ATP synthase
ATPS	ATP synthase
CITL	citrate lyase
CODH	carbon monoxide dehydrogenase/ acetyl-CoA synthase
CS	Citrate synthase
FDH	formate dehydrogenase
FDXMQpp	Ferredoxin-dependent oxidorectase
FHL	formate hydrogen lyase
FRD2	fumarate dehydratase
FTHFD	formyl-TFH deformylase
FUM	fumarate hydratase
GLUDy	glutamate dehydrogenase
H2CYTBr	cytochrome b dependent hydrogenase
HYD-NADH	NADH dependent hydrogenase
HYDA_Ech	Ech-type hydrogenase
HYDFDN2	electron bifurcating hydrogenase
ICDHyr	isocitrate dehydrogenase
ME2	malic enzyme
MecEBF	Mec cassette
METR	5-methyl-THF corrinoid:FeS protein methyltransferase
MTHFC	methenyl-THF cyclohydrolase
MTHFD	methylene-THF dehydrogenase
MTHFR	methylene-THF reductase
OORr	oxoglutarate oxidoreductase
PC	pyruvate carboxylase
POR	pyruvate oxidoreductase
RDase	reductive dehalogenase
STN	*Sporomusa*-type Nfn
SUCOAS	succinate CoA ligase

**Code**	**Metabolite**
OAA	oxaloacetate
CIT	citrate
ICIT	isocitrate
AKG	alpha-ketoglutarate
SUCC	succinate
FUM	fumarate
MAL	malate
THF	tetrahydrofolate
fdx	ferredoxin
mql	menaquinol
mqn	menaquinone
PYR	pyruvate

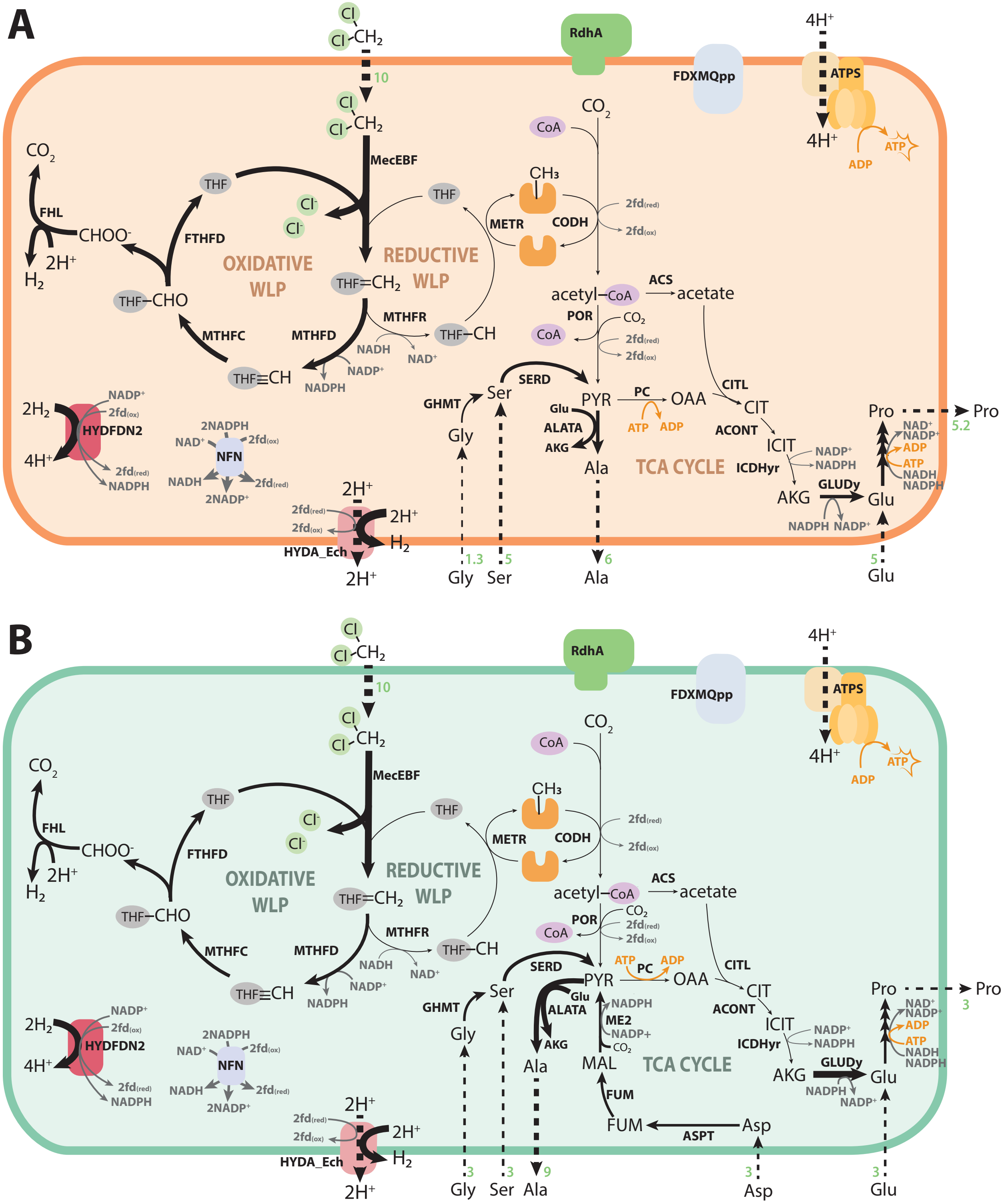

Figure S4. Flux distribution during growth **Mode 3, Strategy A**, using DCM as an electron donor and H^+ as an electron acceptor, with minimal amino acid supplementation. **A**) *Dehalobacter* strain SAD, **B**) *Dehalobacter* strain DAD. Amino acid exchange flux values are shown in green (mmol gdw^{-1} d^{-1}), arrows are scaled to relative flux magnitude. Enzyme/ metabolite codes are listed in Section 3.1.

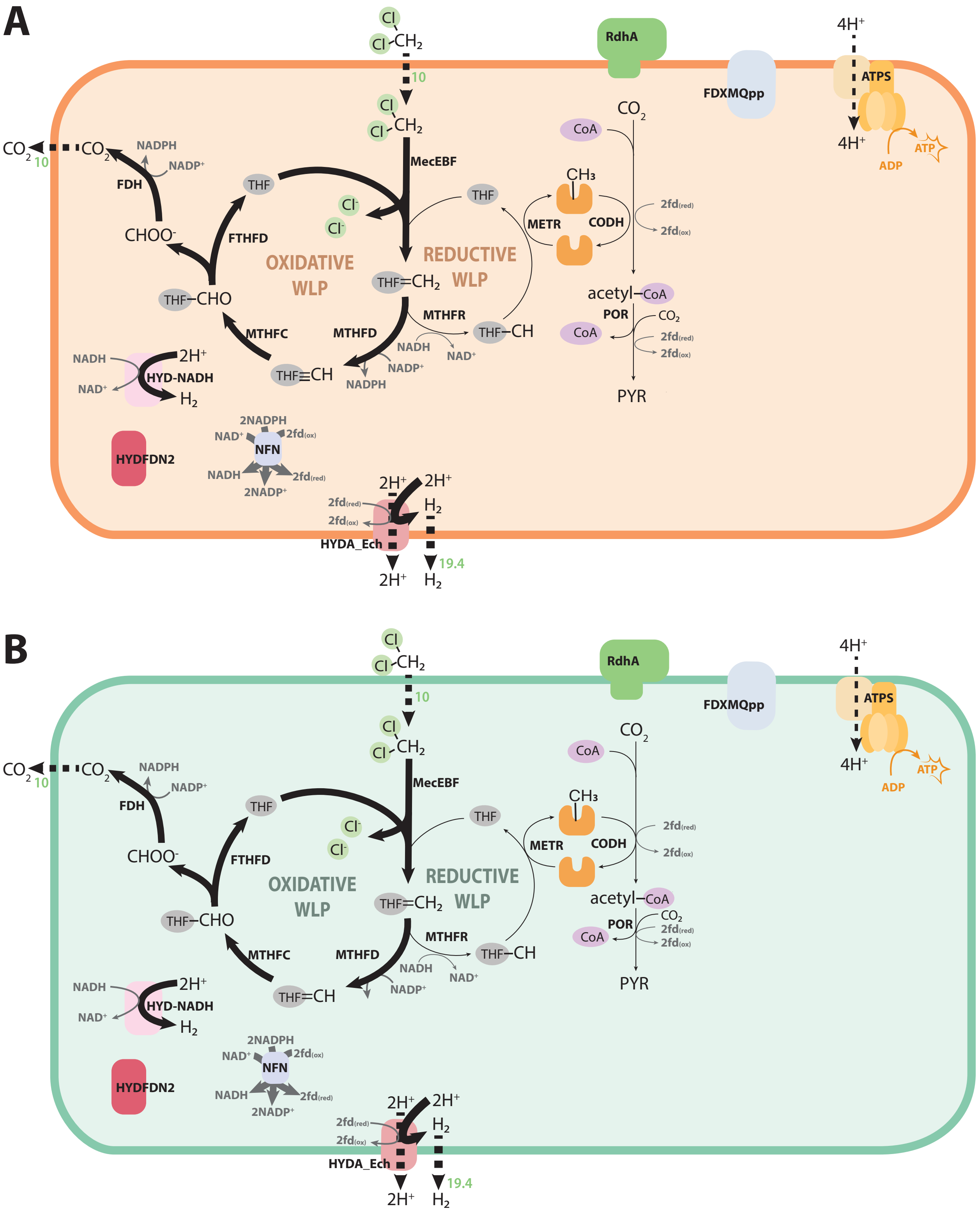

Figure S5. Flux distribution during growth **Mode 3, Strategy B**, using DCM as an electron donor and H^+ as an electron acceptor, with addition of an NADH-dependent hydrogenase HYD-NADH. **A**) *Dehalobacter* strain SAD, **B**) *Dehalobacter* strain DAD. Exchange flux values shown in green, arrows are scales to relative flux. Enzyme/metabolite codes are listed in Section 3.1.

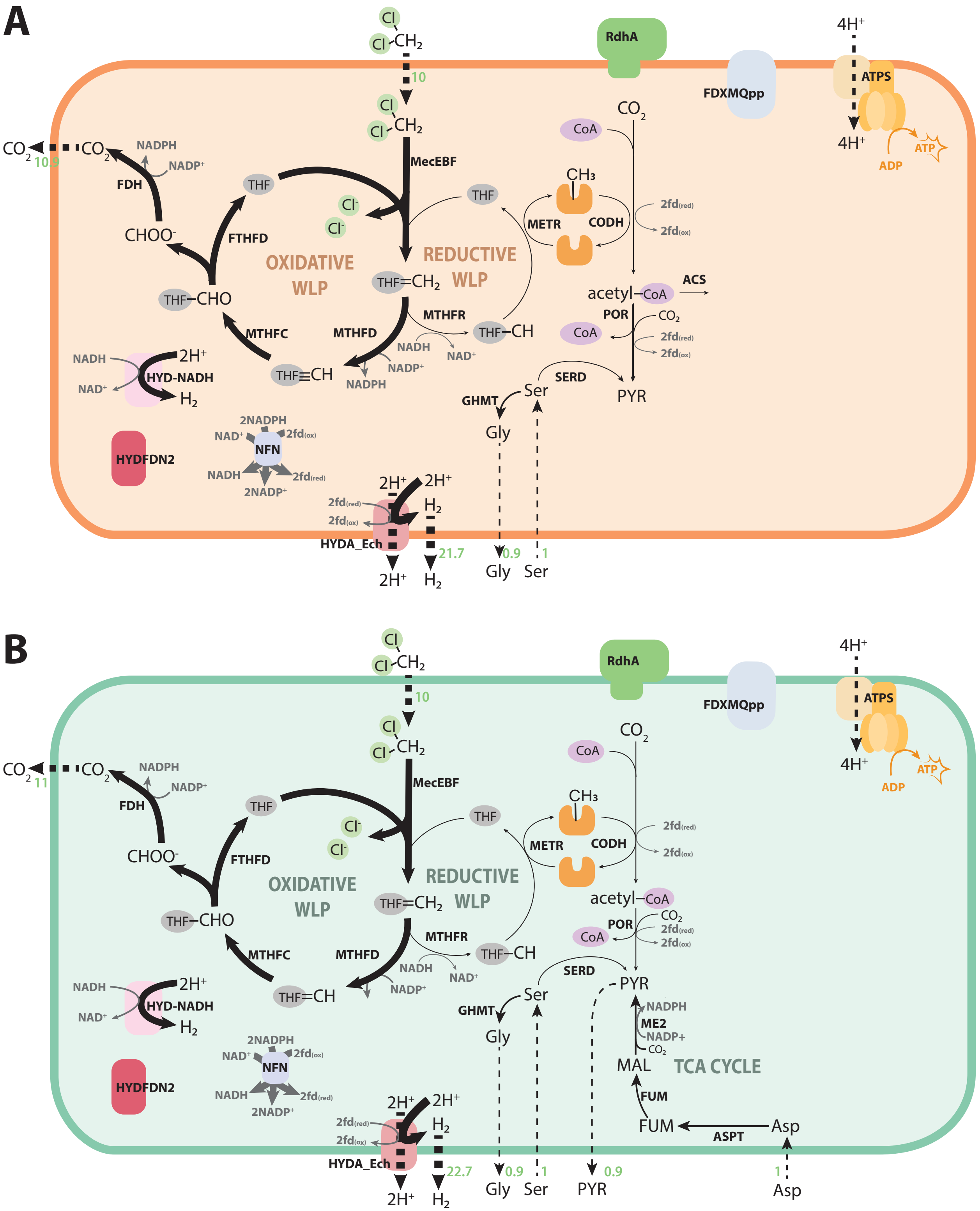

Figure S6. Flux distribution during growth **Mode 3, Strategy C**, using DCM as an electron donor and H^+ as an electron acceptor, with addition of an NADH-dependent hydrogenase HYD-NADH and minimal amino acid supplementation. **A**) *Dehalobacter* strain SAD, **B**) *Dehalobacter* strain DAD. Exchange flux values shown in green, arrows are scales to relative flux. Enzyme/metabolite codes are listed in Section 3.1.

3.2. The impact of product inhibition on H_2-evolving hydrogenases

Sub-transfers of SC05-UT are often amended with hydrogen at the beginning of time course experiments to jump-start dechlorination, or when decoupling from DCM degradation is desired to disentangle yield calculations. Despite its overall thermodynamic feasibility at partial pressures up to 1 atm H_2, DCM mineralization is known to stall due to hydrogen inhibition at lower partial pressures (40–42). This has been attributed to the product inhibition of hydrogen-evolving [NiFe] and [FeFe] hydrogenases (43, 44). To simulate the *Dehalobacter* metabolism when amended with hydrogen, including its deleterious effects on hydrogenases, all H_2 evolving reactions were blocked during FBA simulations of each mode (**Table S11**). Formate hydrogen lyase can produce H_2 without inhibition at high H_2 partial pressures (an order of magnitude higher than inhibits other [NiFe] hydrogenases (43)) thus was not constrained.

Indeed, when H_2 evolution was inhibited, biomass could not be produced from DCM (Mode 3) in either strain, even when supplied with amino acids. When CF and H_2 were provided to the model under H_2 evolution inhibition (Mode 1), only 12-50% of DCM was assimilated. This suggests DCM assimilation is not favored without hydrogen production. Experimentally, <30% of DCM typically accumulates when H_2 is provided to the culture.

During Mode 1 under H_2 evolution inhibition, strain SAD uses a Group 1a [NiFe] H_2-uptake hydrogenase (H2CYTb) to reduce cytochrome b, subsequently reducing menaquinone to menaquinol (**Figure S7A**). Menaquinol donates electrons to the RDase to dechlorinate CF, consistent with non-DCM mineralizing *Dehalobacter* strains (16, 33). In the absence of HYDA_Ech, strain SAD relies on H2CYTb to split H_2 in the periplasm and menaquinone shuttling to establish a proton gradient.

In strain DAD, a Group A3 [Fe-Fe] hydrogenase (HYDFDN2) is the main hydrogen uptake mechanism. This hydrogenase reduces ferredoxins, which are transferred to FDXMQpp. Protons are translocated by FDXMQpp, producing menaquinol to reduce the reductive dehalogenase. Strain DAD also uses FRD2 to produce menaquinol, accounting for its slightly higher growth rate (**Figure S7B**). Flux though FRD2 and ASPT facilitates redox balancing and increases production of biomass (**Table S11**).

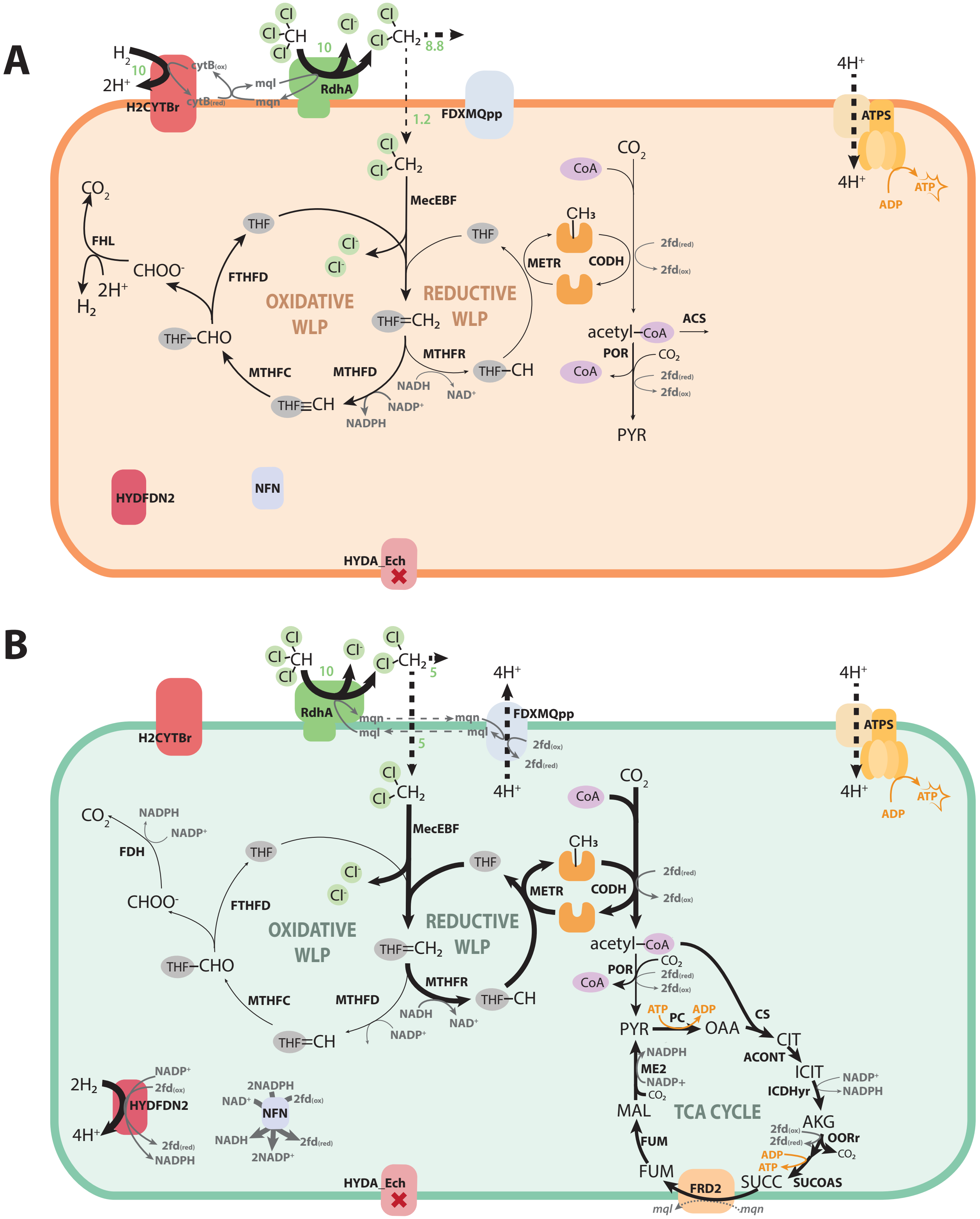

Figure S7. Flux distribution during growth **Mode 1, Strategy A, H_2 evolution inhibition**, using H_2 as an electron donor and CF as an electron acceptor. A) *Dehalobacter* strain SAD, B) *Dehalobacter* strain DAD. Flux values shown in green, arrows are scales to relative flux. Red X shows inhibited reaction. Enzyme/metabolite codes are listed in Section 3.1.

3.3. Yield validation of different modes

Model predicted yields were calculated by dividing biomass flux by electron donor flux and converting to eeq/mol. The model predicted yields in each mode and condition were compared to the experimentally calculated yields in Table 4.

Experimentally, 70-100% of DCM accumulates during Mode 1 while H_2 is provided as an electron donor, which is most consistent with the predications of simulation cases B and C (**Table S11**). Though we have not attempted to measure hydrogen production in SC05-UT or DCME, hydrogen accumulation was detected by Wang et al. in the parent culture, SC05, growing under Modes 2 and 3, wherein 1.5% and 9% of electrons from DCM were recovered as hydrogen, respectively (40). Hydrogen export is only predicted during Strategies B and C, when HYDFDN is inhibited, and higher ratios of hydrogen to DCM are predicted than measured experimentally (**Table S11**).

During Strategy B, Modes 2 and 3 export 47-50% and 97% of electrons from DCM as hydrogen, respectively, which indicates that very few electrons are going to biomass in Mode 3 and explains the low growth yields. When supplied additional amino acids, Modes 2 and 3 export more hydrogen, due to import and oxidation of amino acids, but even without supplied amino acids, the estimated hydrogen production is higher than what has been measured experimentally. This is due to the microbial community in SC05, which comprises many hydrogen-consuming microorganisms like methanogens and acetogens that deplete accumulating hydrogen. Overall, Strategy C is likely to occur in the SC05-UT and DCME cultures, based on growth yield comparisons and experimental hydrogen production and DCM accumulation.

3.4. Role of methanogens in SC05

At low hydrogen concentrations, CF dechlorination is more thermodynamically favourable than methanogenesis; as the ΔG of methanogenesis becomes positive at the µM level, the ΔG of CF dechlorination remains highly negative (Figure S8), allowing dechlorination to occur at lower concentrations of external hydrogen than methanogenesis. Moreover, CF inhibits growth of most methanogens, thus must be converted to DCM in full before methanogenesis can occur (**Figure S8**). Though DCM can also inhibit methanogenesis (45–47), higher concentration (>2 mM for partial inhibition (48)) are required and this effect is not seen in SC05, as DCM concentrations rarely exceed 1 mM.

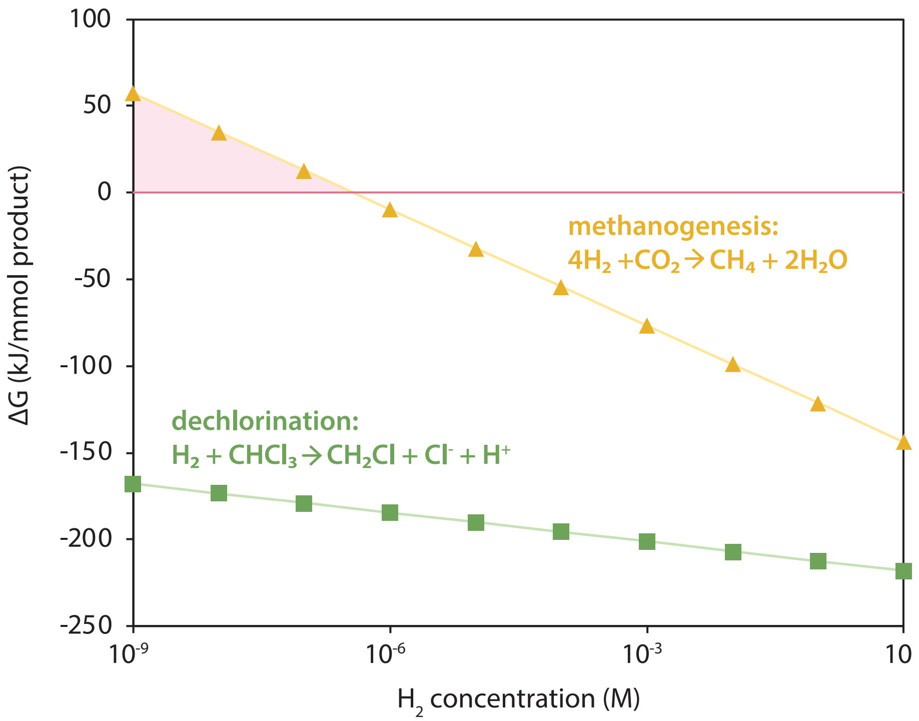

Figure S8. Thermodynamics of CF dechlorination and methanogenesis at various H_2 concentrations. Hydrogen concentrations prohibiting methanogenesis are highlighted in pink.

References for SI

1. Correia K, Ho H, Mahadevan R. 2018. Genome-scale metabolic network reconstruction of the chloroform-respiring *Dehalobacter restrictus* strain CF. bioRxiv https://doi.org/10.1101/375063. DOI:10.1101/375063.

2. Dauner M, Storni T, Sauer U. 2001. Bacillus subtilis Metabolism and Energetics in Carbon-Limited and Excess-Carbon Chemostat Culture. J Bacteriol 183:7308–7317. DOI:10.1128/jb.183.24.7308-7317.2001.

3. Holliger C, Hahn D, Harmsen H, Ludwig W, Schumacher W, Tindall B, Vazquez F, Weiss N, Zehnder AJB. 1998. *Dehalobacter restrictus* gen. nov. and sp. nov., a strictly anaerobic bacterium that reductively dechlorinates tetra- and trichloroethene in an anaerobic respiration. Arch Microbiol 169:313–321. DOI:10.1007/s002030050577.

4. Neidhardt FC, Ingraham JL, Schaechter M. 1990. Physiology of the bacterial cell: a molecular approach. Sinauer Associates, Inc, Sunderland, Massachusetts.

5. Henry CS, Zinner JF, Cohoon MP, Stevens RL. 2009. iBsu1103: A new genome-scale metabolic model of *Bacillus subtilis* based on SEED annotations. Genome Biol 10:1–15. DOI:10.1186/gb-2009-10-6-r69.

6. Feist AM, Scholten JCM, Palsson B, Brockman FJ, Ideker T. 2006. Modeling methanogenesis with a genome-scale metabolic reconstruction of *Methanosarcina barkeri*. Mol Syst Biol 2:2006.0004. DOI:10.1038/msb4100046.

7. Pramanik J, Keasling JD. 1997. Stoichiometric model of Escherichia coli metabolism: Incorporation of growth-rate dependent biomass composition and mechanistic energy requirements. Biotechnol Bioeng 56:398–421. DOI:10.1002/(sici)1097-0290(19971120)56:4<398::aid-bit6>3.0.co;2-j,.

8. Pramanik J, Keasling JD. 1998. Effect of Escherichia coli biomass composition on central metabolic fluxes predicted by a stoichiometric model. Biotechnol Bioeng 60:230–238. DOI:10.1002/(sici)1097-0290(19981020)60:2<230::aid-bit10>3.0.co;2-q,.

9. Mahadevan R, Bond DR, Butler JE, Esteve-Nuñez A, Coppi M V., Palsson BO, Schilling CH, Lovley DR. 2006. Characterization of Metabolism in the Fe(III)-Reducing Organism Geobacter sulfurreducens by Constraint-Based Modeling. Appl Environ Microbiol 72:1558–1568. DOI:10.1128/aem.72.2.1558-1568.2006.

10. Daae EB, Ison AP. 1999. Classification and sensitivity analysis of a proposed primary metabolic reaction network for Streptomyces lividans. Metab Eng 1:153–165. DOI:10.1006/mben.1998.0112.

11. Rittmann BE, McCarty PL. 2001. Chapter 2: Stoichiometry and Bacterial Energetics, p. 126–166. *In* Environmental Biotechnology: Principles and Applications.

12. Kröger A, Biel S, Simon J, Gross R, Unden G, Lancaster CRD. 2002. Fumarate respiration of *Wolinella succinogenes*: Enzymology, energetics and coupling mechanism. Biochim Biophys Acta 1553:23–38. DOI:10.1016/s0005-2728(01)00234-1.

13. Thauer RK, Jungermann K, Decker K. 1977. Energy conservation in chemotrophic anaerobic bacteria. Bacteriol Rev 41:100–180. DOI:10.1128/mmbr.41.1.100-180.1977.

14. Rittmann BE, McCarty PL. 2001. Environmental biotechnology: principles and applications. Curr Opin Biotechnol 7:357–365. DOI:10.1016/s0958-1669(96)80047-4.

15. Dolfing J;, Janssen DB. 1994. Estimates of Gibbs free energies of formation of chlorinated aliphatic compounds. Biodegradation 5:21–28.

16. Schumacher W, Holliger C. 1996. The proton/electron ratio of the menaquinone-dependent electron transport from dihydrogen to tetrachloroethene in "*Dehalobacter restrictus*." J Bacteriol 178:2328–2333. DOI:10.1128/jb.178.8.2328-2333.1996.

17. Turina P, Samoray D, Gräber P. 2003. H+/ATP ratio of proton transport-coupled ATP synthesis and hydrolysis catalysed by CF0F1-liposomes. EMBO J 22:418–426. DOI:10.1093/emboj/cdg073.

18. Adrian L, Löffler FE. 2016. Organohalide-Respiring Bacteria. Springer Nature, Berlin Heidelberg.

19. Islam MA, Edwards EA, Mahadevan R. 2010. Characterizing the Metabolism of *Dehalococcoides* with a Constraint-Based Model. PLoS Comput Biol 6:e1000887. DOI:10.1371/journal.pcbi.1000887.

20. Guo S, Toth CRA, Luo F, Chen X, Xiao J, Edwards EA. 2022. Transient Oxygen Exposure Causes Profound and Lasting Changes to a Benzene-Degrading Methanogenic Community. Environ Sci Technol 56:13036–13045. DOI:10.1021/acs.est.2c02624.

21. Bulka O, Picott K, Mahadevan R, Edwards EA. 2024. From *mec* cassette to *rdhA*: a key *Dehalobacter* genomic neighborhood in a chloroform and dichloromethane–transforming microbial consortium. Appl Environ Microbiol 90:1–24. DOI:10.1128/aem.00732-24.

22. Bulka O, Mahadevan R, Edwards EA. 2024. Pangenomic insights into Dehalobacter evolution and acquisition of functional genes for bioremediation. Microb Genomics 10:001324. DOI:10.1099/mgen.0.001324.

23. Willemin MS, Armand F, Hamelin R, Maillard J, Holliger C. 2024. Conditional essentiality of the 11-subunit complex I-like enzyme in strict anaerobes: the case of Desulfitobacterium hafniense strain DCB-2. Front Microbiol 15:1388961. DOI:10.3389/fmicb.2024.1388961/bibtex.

24. Søndergaard D, Pedersen CNS, Greening C. 2016. HydDB: A web tool for hydrogenase classification and analysis. Sci Rep 6:34212. DOI:10.1038/srep34212.

25. Greening C, Biswas A, Carere CR, Jackson CJ, Taylor MC, Stott MB, Cook GM, Morales SE. 2015. Genomic and metagenomic surveys of hydrogenase distribution indicate H2 is a widely utilised energy source for microbial growth and survival. ISME J 2016 103 10:761–777. DOI:10.1038/ismej.2015.153.

26. Bulka O. 2024. Dataset S3: Proteomic statistical analysis and identification of peptides and proteins from microbial consortia SC05-UT and DCME. figshare https://doi.org/10.6084/m9.figshare.25146029.v1. DOI:10.6084/m9.figshare.25146029.v1.

27. Rupakula A, Kruse T, Boeren S, Holliger C, Smidt H, Maillard J. 2013. The restricted metabolism of the obligate organohalide respiring bacterium *Dehalobacter restrictus*: Lessons from tiered functional genomics. Philos Trans R Soc B Biol Sci 368:20120325. DOI:10.1098/rstb.2012.0325.

28. Kremp F, Roth J, Müller V. 2020. The *Sporomusa* type Nfn is a novel type of electron-bifurcating transhydrogenase that links the redox pools in acetogenic bacteria. Sci Rep 10:14872. DOI:10.1038/s41598-020-71038-2.

29. Huang H, Wang S, Moll J, Thauer RK. 2012. Electron bifurcation involved in the energy metabolism of the acetogenic bacterium *Moorella thermoacetica* growing on glucose or H2 plus CO2. J Bacteriol 194:3689–3699. DOI:10.1128/jb.00385-12.

30. Gilchrist CLM, Chooi YH. 2021. Clinker & clustermap.js: Automatic generation of gene cluster comparison figures. Bioinformatics 37:2473–2475. DOI:10.1093/bioinformatics/btab007.

31. Tang S, Wang PH, Higgins SA, Löffler FE, Edwards EA. 2016. Sister *Dehalobacter* genomes reveal specialization in organohalide respiration and recent strain differentiation likely driven by chlorinated substrates. Front Microbiol 7:1–14. DOI:10.3389/fmicb.2016.00100.

32. Anantharaman V, Aravind L. 2002. MOSC domains: ancient, predicted sulfur-carrier domains, present in diverse metal-sulfur cluster biosynthesis proteins including molybdenum cofactor sulfurases. FEMS Microbiol Lett 207:55–61. DOI:10.1111/j.1574-6968.2002.tb11028.x.

33. Wang P-HH, Correia K, Ho H-CC, Venayak N, Nemr K, Flick R, Mahadevan R, Edwards EA. 2019. An interspecies malate–pyruvate shuttle reconciles redox imbalance in an anaerobic microbial community. ISME J 13:1042–1055. DOI:10.1038/s41396-018-0333-4.

34. Maphosa F, Van Passel MWJ, De Vos WM, Smidt H. 2012. Metagenome analysis reveals yet unexplored reductive dechlorinating potential of *Dehalobacter* sp. E1 growing in co-culture with *Sedimentibacter* sp. Environ Microbiol Rep 4:604–616. DOI:10.1111/j.1758-2229.2012.00376.x.

35. Mägli A, Wendt M, Leisinger T. 1996. Isolation and characterization of *Dehalobacterium formicoaceticum* gen. nov. sp. nov., a strictly anaerobic bacterium utilizing dichloromethane as source of carbon and energy. Arch Microbiol 166:101–108. DOI:10.1007/s002030050362.

36. Mägli A, Messmer M, Leisinger T. 1998. Metabolism of dichloromethane by the strict anaerobe *Dehalobacterium formicoaceticum*. Appl Environ Microbiol 64:646–650. DOI:10.1128/aem.64.2.646-650.1998.

37. Murdoch RW, Chen G, Murdoch FK, Mack EE, Villalobos Solis MI, Hettich RL, Löffler FE, Kara Murdoch F, Mack EE, Villalobos Solis MI, Hettich RL, Löffler FE. 2022. Identification and widespread environmental distribution of a gene cassette implicated in anaerobic dichloromethane degradation. Glob Chang Biol 28:2396–2412. DOI:10.1111/gcb.16068.

38. Wasmund K, Trueba-Santiso A, Vicent T, Adrian L, Vuilleumier S, Marco-Urrea E. 2023. Proteogenomics of the novel *Dehalobacterium formicoaceticum* strain EZ94 highlights a key role of methyltransferases during anaerobic dichloromethane degradation. Environ Sci Pollut Res 1:1–11. DOI:10.1007/s11356-023-28144-1.

39. Wang PH, Tang S, Nemr K, Flick R, Yan J, Mahadevan R, F Yakunin A, Löffler FE, Edwards EA. 2017. Refined experimental annotation reveals conserved corrinoid autotrophy in chloroform-respiring *Dehalobacter* isolates. ISME J 11:626–640. DOI:10.1038/ismej.2016.158.

40. Wang H, Yu R, Webb J, Dollar P, Freedman DL. 2022. Anaerobic biodegradation of chloroform and dichloromethane with a *Dehalobacter* enrichment culture. Appl Environ Microbiol 88:e01970-21. DOI:10.1128/aem.01970-21.

41. Chen G, Fisch AR, Gibson CM, Erin Mack E, Seger ES, Campagna SR, Löffler FE. 2020. Mineralization versus fermentation: evidence for two distinct anaerobic bacterial degradation pathways for dichloromethane. ISME J 14:959–970. DOI:10.1038/s41396-019-0579-5.

42. Bulka O, Webb J, Dworatzek S, Mahadevan R, Edwards EA. 2023. A multifunctional *Dehalobacter*? Tandem chloroform and dichloromethane degradation in a mixed microbial culture. Environ Sci Technol 57:19912–19920. DOI:10.1021/acs.est.3c06686.

43. McDowall JS, Murphy BJ, Haumann M, Palmer T, Armstrong FA, Sargent F. 2014. Bacterial formate hydrogenlyase complex. Proc Natl Acad Sci U S A 111:E3948–E3956. DOI:10.1073/pnas.1407927111.

44. Lubitz W, Ogata H, Rüdiger O, Reijerse E. 2014. Hydrogenases. Chem Rev 114:4081–4148. DOI:10.1021/cr4005814.

45. Stuckey DC, Owen WF, McCarty PL, Parkin GF. 1980. Anaerobic Toxicity Evaluation by Batch and Semi-Continuous Assays. J Water Pollut Control Fed 52:720–729.

46. Vargas C, Ahlert RC. 1987. Anaerobic Degradation of Chlorinated Solvents. J Water Pollut Control Fed 59:964–968.

47. Thiel PG. 1969. The effect of methane analogues on methanogenesis in anaerobic digestion. Water Res 3:215–223. DOI:10.1016/0043-1354(69)90060-8.

48. Freedman DL, Gossett JM. 1991. Biodegradation of dichloromethane and its utilization as a growth substrate under methanogenic conditions 57:2847–2857. DOI:10.1128/aem.57.10.2847-2857.1991.
